# Social Media Big Data: The Good, The Bad, and the Ugly (Un)truths

**DOI:** 10.3389/fdata.2021.623794

**Published:** 2021-06-01

**Authors:** Alton M. K. Chew, Dinesh Visva Gunasekeran

**Affiliations:** ^1^ Yong Loo Lin School of Medicine, National University of Singapore (NUS), Singapore, Singapore; ^2^ UCL Medical School, University College London (UCL), London, United Kingdom

**Keywords:** coronavirus—COVID-19, public health, big data, social media, health promotion

## Introduction

Social media has been a defining component of life in the 21st century, monetising peer-to-peer sharing of information. This has led to the formation of powerful platforms leveraging artificial intelligence (AI) to effectively commoditise individual attention, with the average person spending over 2 h a day on social media (Statista, 2020). The ubiquity of these platforms underscores the need to better understand social media in the context of public health, in particular its potential risks and benefits. This will facilitate comprehensive assessment of its impact on healthcare services, development of mitigating strategies for its drawbacks, and identification of potential opportunities to leverage its strengths for the good of population health.

These sentiments were recently echoed by the World Health Organisation (WHO) in the context of the coronavirus disease 2019 (COVID-19) pandemic, calling upon member states to address misinformation through risk communication and timely dissemination of accurate information as a key pillar of national public health responses (World Health Organisation, 2020a). However, our collective experience during this pandemic highlights the limitations of our existing knowledge and approaches, as many countries continue to experience the spiralling social impact of misinformation related to COVID-19. This manuscript provides an overview of both the negative and positive public health impact of social media that has come to light in the course of COVID-19 with an emphasis on rampant disinformation during COVID-19, and concludes with potential future directions for research in this emerging area of public health.

Viral Infodemic in Social Media outpaces the Pandemic in the Streets–the (Un)truths

The COVID-19 outbreak that originated from Wuhan city, China, has already claimed over 1 million lives and infected over 40 million individuals since it was first reported in December 2019. With the initial spread of COVID-19 progressing to pandemic status, the WHO also recognized the parallel problem of widespread anxiety and emotional sharing of information through digital platforms. This imposed an additional strain on humanity’s coordinated attempts to eradicate COVID-19, hindering public health risk communication (The Lancet Infectious Diseases, 2020). Experts have studied big data in online search and social media with many reports highlighting surges in online misinformation in each region even before the uptick in confirmed cases (Hong et al., 2020). Poor quality online sources have drowned out official advisories with fake and potentially harmful information (Cuan-Baltazar et al., 2020). This presents major health concerns given the public’s difficulty in differentiating reliable from unreliable sources of medical information, even among individuals with good baseline health literacy (Huhta et al., 2018).

Further aggravating this situation, the excessive amounts of misinformation interspersed with official sources of information shared across social media platforms has placed a heavy burden on the public to discern true from false information. The WHO has since identified this phenomenon as an “infodemic”, making calls for novel solutions to moderate the flow of accessible information and stem medical disinformation (World Health Organisation, 2020b). A recent survey of social media users in Kurdistan, Iraq further emphasised the negative impact of this problem, whereby the researchers have reported how health-related content featured prominently in social media during COVID-19 with fake news accounting for over a quarter of “fear-inducing” content (Ahmad and Murad, 2020). This study was not appropriately designed to evaluate the relationship between social media content and fear, which was also inflated by selection bias. However, it does highlight the pervasiveness of this problem particularly considering the prominence of social media in modern life. This begets the need for solutions to adjudicate content in social media, and provide a check and balance to the spread of misinformation via these viral platforms.

Social media big data and public health during COVID-19—the Good and the Bad

The extensive usage of social media naturally includes large amounts of information regarding users’ unadulterated feelings and thoughts documented in a publicly visible platform. The collection of this information provides ‘naturally-occurring’ and publicly-visible big data, that can potentially be applied to improve Public Health responses during Natural disasters and emergencies such as Pandemics. Several such applications of big data have been described during the COVID-19 pandemic, including regular *COVID-19 Snapshot MOnitoring* (*COSMO*) initiated in Germany to improve surveillance of misinformation and inform the development of policies and communication messages (Betsch et al., 2020).

Moreover, researchers from China demonstrated the use of big data from social media platform WeChat to identify trends in communication and searches for key words related to these topics. Using these “infodemiology” techniques, which analyse online user generated content (UGC) to inform public health applications, researchers could correlate digital big data to the progression of the pandemic unfolding in real-time (Lu and Zhang, 2020). By harnessing this readily available online information, researches have further demonstrated “infodemiology” techniques for applications such as planning of pandemic responses, optimising the flow of resources, and identifying growing themes of misinformation and/or public concerns real-time to develop targeted public health strategies and communications (Wong et al., 2020).

### Emerging Research in Social Media Big Data for Public Health Interventions

The reports described in the previous section have illustrated “infodemiology” techniques that leverage big data from social media for timely insights that could inform the development of critical public health responses. Other researchers have further described the amalgamation of multimodal data from social media complemented by other sources such as traditional news media and online behaviour/market research agency platforms to inform the development of evidence-based public health interventions. These capabilities have been demonstrated for the evaluation of the public’s compliance to public health measures as well as the evaluation of national responses to help control the pandemic in China, using data from social media such as Weibo and Tik Tok, the People’s Daily major Chinese newspaper, and online market research agency platforms such as Mob-Tech research institute (Hua and Shaw, 2020).

Researchers have further demonstrated the potential utility of new techniques such as Online Ecological Recognition (OER) that combine big data with other emerging technology domains like artificial intelligence (AI) to develop predictive models (Li S. et al., 2020). This facilitated additional applications beyond the surveillance of information, to evaluating the mental health impact of the pandemic itself. The study demonstrated that negative emotions and sensitivity to social risks increased while positive emotions and life satisfaction plummeted (Li S. et al., 2020). The results from this particular study corroborated with the Behavioural Immune System (BIS) theory that people tend towards negative emotions when threatened by disease, whereby the spike in negative emotion was heightened during COVID-19 due to the infodemic.

However, another study of 17,865 Weibo users in China highlighted a silver lining regarding the impact of social media during this pandemic, whereby initial negative emotions (after COVID-19 was reported widely) were subsequently balanced by positive emotions as users leveraged social media platforms for peer-support, with trending topics such as “faith” and “blessing”. (Li S. et al., 2020). These terms reflect greater group cohesiveness given the threat to greater public, and these findings were further replicated in Lombardy based on data from Italy (Su et al., 2020). Notably, the increased group cohesion occurred in tandem with more monetary and supply donations to regions of need and key organisations including the Hubei Red cross (Li S. et al., 2020). It is thus evident that social media can be leveraged for positive impact, by helping to connect individuals during a crisis and improve individual alignment for common good. This has additional implications for other aspects of medication, including the use of these platforms for health promotion and raising awareness about critical health-related problems (Horrell et al., 2019). Through further investigation and refinement of these methods, public health organisations will be able to optimise response strategies in real-time by extrapolating trends in transmission, communication content, information flow, and population sentiment.

### What Lies Ahead

Our article has highlighted the potential impact of social media big data to be a double-edged sword. Presently, the negative impact has gained much visibility and criticism, due to limited mechanisms for differentiating reliable information from misinformation, and mitigating the risks of the latter. Fortunately, increasing coordination between social media platform providers, non-governmental organisations, and governments have given rise to promising collaborations such as the “Share verified” initiative led by the United Nations (UN) to build a freely-accessible resource of reliable health content and front-end flags to redirect individuals to reliable sources in order to address misinformation. Ultimately, long-term solutions may require new legislation to govern the creation and dissemination of misinformation online. Regulations have been effectively applied for other public health challenges, such as tobacco advertising regulations to reduce population exposure to marketing and cues to smoke (Henriksen, 2012).

However, in the case of online misinformation, the enforcement of such legislation will be significantly more complexed, given the scale of individuals as potential sources as opposed to corporations that are stakeholders within the tobacco industry. This will likely require methods such as COSMO for big data surveillance, with incorporation of AI analytics of the social media big data to scale up enforcement. This also begets consideration of developing alternatives and complements to social media as sources of reliable health information hosting and exchange, with several recently launched in response to COVID-19 misinformation. Online health communities (OHCs) have drawn increasing interest in the domain of virtual social networks due to their potential to amplify positive impact such as peer-support and quality data as a source of health evidence (Smith et al., 2017; Audrain-Pontevia et al., 2019), as well as mitigate against negative impact through policies against the promotion of inaccurate information, as well as configurations that involve medical practitioners in moderation and content generation (Eysenbach, 2000; AskDr, 2020).

However, even with these measures in place, studies have highlighted the potential for lapses to occur that can be difficult to detect (Huh et al., 2016). Therefore, there is a growing need for OHCs that leverage the strengths of social media platforms with additional embodiments that mitigate against its weaknesses. These may be configurations that empower verified medical experts with digital tools to moderate the content and flow of information. These applications of OHCs for patients with chronic pain and mental health disorders that are likely to progress and increase in prevalence during COVID-19 have been described in earlier reviews led by relevant specialist (Chew et al., 2020; Li L. W. et al., 2020). These digital platforms represent potential areas for future research and cross-disciplinary collaborations between technology partners, clinicians and regulators to enhance public health responses.

## Conclusion

Social media has likely been a significant contributor to the dissemination of misinformation and fear in this pandemic, particularly given the lack of information arbitration and controls of viral false information (Li L. W. et al., 2020). However, several applictions of social media big data about health-related content for public health communication measures have been discussed in this study. These studies shed light on the potential positive impact and applications of social media in public health. Much is still unknown, and it would be impossible to weigh the benefits and drawbacks of social media in healthcare at this stage. The only certainty is that social media is likely to remain a prominent feature of modern life, and more research is needed to better understand this domain to amplify its positive impact and to mitigate against the negative.
